# The emerging sequence grammar of 3D genome organisation

**DOI:** 10.1007/s00439-025-02772-8

**Published:** 2025-08-25

**Authors:** Liezel Tamon, James Ashford, Matthew Nicholls, Marella F.T.R. de Bruijn, Aleksandr B. Sahakyan

**Affiliations:** 1 MRC WIMM Centre for Computational Biology, MRC Weatherall Institute of Molecular Medicine, Radcliffe Department of Medicine, University of Oxford, Oxford, OX3 9DS UK; 2 MRC Molecular Haematology Unit, MRC Weatherall Institute of Molecular Medicine, Radcliffe Department of Medicine, University of Oxford, Oxford, OX3 9DS UK

## Abstract

The multiplexed layers of regulatory processes and mechanisms within a cell are, to a degree, encoded in our genome. Unravelling the relationship between DNA sequence and molecular processes is crucial for understanding evolution, interpreting and predicting the consequences of genomic variation. Furthermore, understanding the extent to which DNA sequence contributes to the genome organisation can help reveal the aspects more influenced by other factors. Therefore, here we provide a succinct summary of the emerging genomic sequence code or “grammar” of genomic contact formation and 3D genome organisation. Drawing on different types of evidence from multiple disciplines, from large-scale genomic studies, biochemical in vitro assays, and computational analyses utilising machine learning and other modelling techniques, we aim to inform future research on the present associations between 3D genome organisation and sequence.

## Introduction

### Multiscale organisation of the 3D genome

The genome within a eukaryotic cell is intricately organised, confining it inside the nucleus, while still ensuring necessary molecular functions. This organisation can be categorised into three scales: (1) nucleosome- and chromatin-fibre scale, (2) intermediate domain-scale (consisting of chromatin loops and other longer-range contacts between regions, topologically-associated domains or TADs, lamin-associated domains or LADs, and chromatin compartments), and (3) nuclear scale (consisting of chromosome territories, and genome arrangements relative to the nuclear centre/periphery and nuclear bodies). At the smallest scale, DNA is packaged into arrays of nucleosomes that are then arranged further into chromatin fibres of 5–24 nm in diameter (Ou et al. 2017). Chromatin regions interact with each other, some constrained within domains like TADs, which consist of regions that preferentially interact with each other. Contacts and chromatin domains are organised into higher-order A and B compartments, generally correlated with the earlier observed self-segregation of euchromatin and heterochromatin, respectively. At the largest scale, chromosomes exist in territories that are non-randomly positioned relative to nuclear bodies (e.g. nucleolus, nuclear speckles and Cajal bodies), depending on the biological activity of these nuclear bodies, and are also non-randomly organised with respect to the nuclear periphery (LADs), and the nuclear centre.

### Mechanisms of 3D genome organisation

A number of mechanisms driving 3D genome organisation have been described, with distinct mechanisms, or combinations of them, operating at different scales (Misteli 2020). Nucleosome positioning is mainly influenced by several factors, as reviewed in (Chereji and Clark 2018): sequence-dependent mechanical properties and energetics determining histone octamer affinity, non-histone proteins (like transcription factors) competing for DNA binding and acting as barriers to create nucleosome-depleted regions, ATP-dependent chromatin remodellers, and transcription. Chromatin fibre configuration, on one hand, can be dependent on DNA linker lengths and post-translational modifications of histones, as reviewed in (Ozer et al. 2015). At the higher-order scales, chromatin fibres interact leading to stochastic contacts, connecting regulatory elements, enabling coregulation of gene clusters, and facilitating genome compaction, particularly through longer-range interactions. These interactions are mediated by the interplay of the following mechanisms:

1) **Inherent spatiotemporal characteristics of the DNA according to its polymeric molecular nature.** This component has been studied through polymer simulations, often using coarse-grained models that represent DNA as a diffusing, self-avoiding polymer. Those models have explained some of the features of the genome architecture, as reviewed in (Rosa and Zimmer 2014).

2) **Domain-forming loop extrusion.** Loop extrusion relies on the DNA binding and extruding activity of factors and the demarcating role of insulator-binding proteins (Sanborn et al. 2015; Fudenberg et al. 2016, 2017). Here, an energy-consuming motor complex, usually cohesin (Davidson et al. 2019) or condensin complex (Gibcus et al. 2018; Ganji et al. 2018), binds to and actively extrudes chromatin, segregates domains and promotes intra-TAD interactions, up until they fall off, or get constrained by insulators, such as CCCTC-binding factor (CTCF), or transcription. These findings are supported by experiments perturbing cohesin complex components and cofactors (Haarhuis et al. 2017; Rao et al. 2017; Schwarzer et al. 2017; Wutz et al. 2017), and by biochemical reconstitution and single-molecule imaging studies (Davidson et al. 2016, 2019; Hansen et al. 2017; Murayama et al. 2018; Gibcus et al. 2018; Ganji et al. 2018).

3) **Self-aggregation of regions and domains of similar type.** This contributes to the formation of higher-order chromosome territories and compartments, within and spanning chromosomes, mainly attributed to phase separation of chromatin-bound proteins, including transcription factors. Polymer simulations (Jost et al. 2014; Nuebler et al. 2018; Falk et al. 2019; Mirny et al. 2019) and in vitro demonstrations of condensate formation by transcription factors and other chromatin-bound proteins, as reviewed in (Hildebrand and Dekker 2020), have shed light on this phenomenon.

4) **Constraints imposed by interactions with the nuclear lamina and other nuclear bodies.** These are associated with the non-random organisation of regions relative to the nuclear periphery (lamina) and centre (Crosetto and Bienko 2020), as well as to nuclear bodies, such as nucleolus, nuclear speckles and Cajal bodies, with the positioning of regions linked to the biological activity of these bodies (Wang et al. 2016; Quinodoz et al. 2018; Chen et al. 2018b). Consistent with the preferential clustering of regions of the same type, similar regions also associate with the nuclear lamina and nuclear bodies. For instance, regions interacting with the lamina are typically transcriptionally inactive, while those co-localised with nuclear speckles are active.

The interplay of mechanisms is highly evident at the level of chromatin loops, with loop extruders like cohesin constraining the regions that can favourably interact (Hansen et al. 2017), and contacts occurring throughout the course of the Brownian motion of DNA (Chen et al. 2018a; Khanna et al. 2019). These contacts are then stabilised by the formation of phase-separated condensates of proteins, including CTCF, transcription factors and chromatin-bound proteins (Sabari et al. 2018; Khanna et al. 2019; Gibson et al. 2019). Even cohesin has been recently shown to phase-separate with DNA in yeast (Ryu et al. 2021). Early studies have shown that weakening cohesin-mediated TAD formation causes the opposing effect of strengthening compartment segregation, indicating that these features are mediated by distinct mechanisms that counteract each other (Rao et al. 2017; Schwarzer et al. 2017; Wutz et al. 2017). Simulation models combining both loop extrusion and phase separation mechanisms have been shown to recapitulate experimental chromatin organisation data at both bulk and single-cell viewpoints (Nuebler et al. 2018; Conte et al. 2022).

### Stochasticity and dynamics of 3D genome organisation and function

Advancements in single-cell, live-cell, and real-time technologies have demonstrated that genome organisation is dynamic and stochastic, revealing extensive heterogeneity across various dimensions, e.g. across cell cycle (Naumova et al. 2013; Nagano et al. 2017), cell types (Schmitt et al. 2016), differentiation stages (Torre-Ubieta et al. 2018; Paulsen et al. 2019), across individual cells (Nagano et al. 2013), and even down to individual alleles (Bintu et al. 2018; Finn et al. 2019; Su et al. 2020). The stochastic nature of 3D genome architecture mirrors gene expression, which is driven by stochastic transcriptional bursts (Elowitz et al. 2002), and the connection between the two remains extensively investigated, as reviewed in (Misteli and Finn 2021; Bohrer and Larson 2021; Sood and Misteli 2022). Early studies hinted at a link by showing that changes in the spatial positioning of genomic regions correlate with changes in cell identity (reviewed in (Bonora et al. 2014)), and that disruptions in TADs can lead to aberrant gene expression (Lupiáñez et al. 2015; Guo et al. 2015). Subsequent work explored this further *via* disruptions of the proposed drivers of organisation, resulting in a wide range of effects, where some cause disease phenotypes, as reviewed in (Krumm and Duan 2019), while others have little (Nora et al. 2017; Rao et al. 2017; Despang et al. 2019) to no (Ghavi-Helm et al. 2019; Williamson et al. 2019) impact. Consequently, chromatin architecture is proposed to function as a structural framework or scaffold that helps to regulate gene expression, with the extent of this control varying depending on specific contexts such as gene identity, location, and the scale of organisation. Progress in this area has been driven by the identification of factors influencing the genome architecture, including the polymeric nature of DNA, complex chromatin information encoded in DNA sequences, epigenetic modifications, and biological processes. These insights come from a combination of biochemical mapping, imaging, polymer modelling, and other computational analyses (Misteli 2020).

### Scope and aim of this review

Beyond carrying genetic information, the genome influences biological processes through its chemical structure, molecular and biophysical characteristics, dynamics, and potential for local conformational changes (e.g. forming secondary structures), all of which are influenced by its sequence. While many studies have revealed how each of these properties can influence cellular behaviour, we lack a concise summary of all known links between these features of the genome sequence and its 3D structure. Here, we discuss how these primary and secondary derivatives of the genome sequence are associated with the different scales of 3D genome organisation, drawing on a wide range of computational and experimental evidence from multiple studies.

## Sequence code of genome organisation

**Fig. 1:**
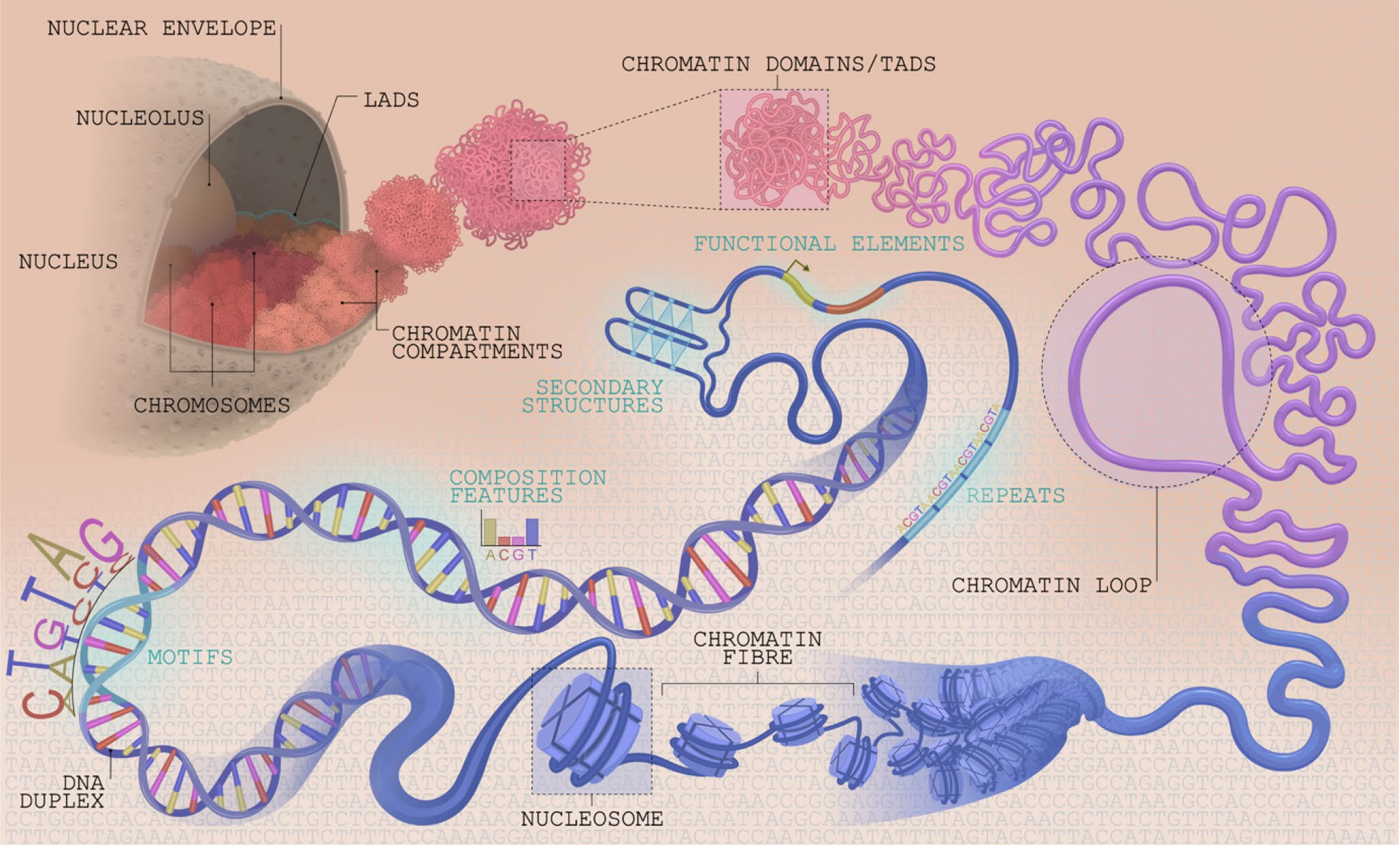
Sequence elements as partakers of the multiscale genome organisation in eukaryotes. The figure schematically depicts the broad categories of sequence features linked to various levels of genome organisation (not at scale), from DNA double helical (and other) structures to nucleosome packing and chromatin fibre compaction, chromatin loops, domains and compartments, including topologically-associated domains (TADs), to nuclear-level organisation with chromosome territories and spatial arrangement relative to nuclear bodies and periphery

**Fig. 2:**
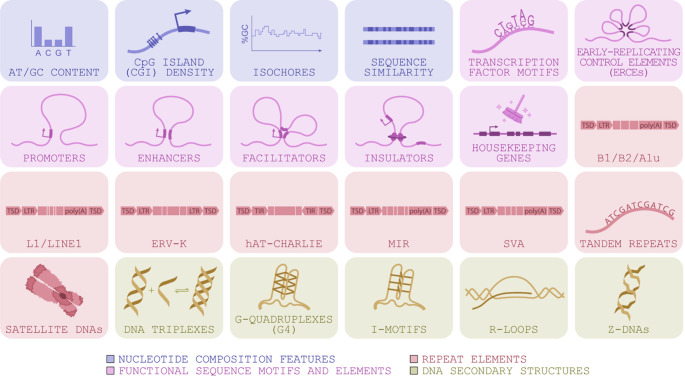
Sequence features associated with 3D genome organisation, grouped according to the broad categories illustrated in Fig. 1

As our understanding of the roles of proteins and epigenetic factors in genome organisation has deepened, researchers have subsequently focused on explaining how this is encoded in the DNA sequence. Insights have emerged from different sources including in vitro experiments, studies that associate 3D genome structural patterns with sequence features, and the development of statistical models used to determine the sequence features important for predicting genome organisation (Yang and Ma 2022). Below, we review the emerging DNA sequence grammar of 3D genome organisation, classifying sequence features into four broad categories: (1) nucleotide composition features, (2) functional sequence motifs and elements, (3) repeat elements, and (4) DNA secondary structures, as illustrated in Fig. 1. Features belonging to each category are enumerated in Fig. 2.

### Nucleotide composition features

Poly(dA:dT) tracts, consisting of stretches of consecutive adenine (A) or thymine (T) bases, are typically depleted of nucleosomes due to their relative rigidity (Segal and Widom 2009), whereas guanine (G) and cytosine (C)-rich sequences generally promote nucleosome occupancy (Tillo and Hughes 2009). Nucleosome-bound sequences display a periodic pattern of dinucleotide sequences (e.g. AA/AT/TA/TT), which influences DNA curvature, observed across different species, e.g. human (Gaffney et al. 2012), yeast (Segal et al. 2006), *Drosophila* (Mavrich et al. 2008), and chicken (Satchwell et al. 1986). Overall, DNA sequence alone is a major influencer of nucleosome positioning, but other factors are at play, including the activity of chromatin remodellers (reviewed in (Chereji and Clark 2018)). At the sub-megabase scale, the nucleotide composition along the genome has been suggested to influence the formation of loops by determining the bendability, supercoiling, and nucleosome density across the genome, with highly GC-rich areas often forming the tips of loops (Naughton et al. 2013; Bernardi 2018). At larger organisation scales, similar sequences generally tend to cluster in space, mirroring the long-observed spatial separation of GC-rich euchromatin (40.9% average GC content in humans (Piovesan et al. 2019)) and AT-rich heterochromatin (Bickmore and van Steensel 2013). Self-segregation of the GC-poor and GC-rich isochore superfamilies and finer families have been found to correspond to compartments and finer subcompartments, respectively (Jabbari and Bernardi 2017; Bernardi 2017; Jabbari et al. 2019). The degree of segregation and intermingling of regions with high and low-CGI or CpG island density (~ 24,000 to 29,000 unique CGIs depending on the method, ~ 1 kb (kilobase) in length in humans (Illingworth and Bird 2009)) was also shown to distinguish different cell types and contexts, leading to proposed phase separation mechanisms of genome organisation informed by sequence and thus not only relying on cell-type-specific epigenomic profiles (Liu et al. 2018). Earlier in vitro experiments have shown evidence of the protein-independent preferential interaction of identical DNA duplexes (termed “DNA self-assembly”) with the help of cations of biologically plausible type and concentration (Kornyshev and Leikin 2001; Inoue et al. 2007; Baldwin et al. 2008; Danilowicz et al. 2009; Yoo et al. 2016; Ohyama 2019), even when the DNA is wrapped in nucleosomes (termed as “nucleosome self-assembly”) (Nishikawa and Ohyama 2013; Zinchenko et al. 2018). Recent work has also linked sequence similarity to persistence of genomic contacts across cell types and tissues using complementarity measures based on k-mers, global alignment and estimation of hybridisation energies (Tamon et al. 2025). With repetitive elements mostly representing similar sequences, it was proposed that repeats mediate a sequence-dependent phase separation of the genome based on this DNA self-assembly phenomenon (Tang 2017). The association of regions may also depend on nucleotide composition. Using molecular dynamics simulations and in vitro experiments, studies have shown that AT-rich sequences interact more favourably than GC-rich ones (Yoo et al. 2016, p. 21; Kang et al. 2018). In a molecular dynamics (MD) simulation, AT-rich molecules spontaneously formed stable bundles, while GC-rich DNA remained dispersed (Kang et al. 2018). Finally, using a deep learning model capable of predicting organisation from kilobase to whole-chromosome scales from DNA sequence (Zhou 2022), it was proposed that B compartmentalisation, which was not linked to any sequence pattern except for preferring AT-rich regions, could be the “default” state of sequences. Sequences could become A-compartment material by having active transcription start sites (TSS) or gaining H3K27ac marks (Nichols and Corces 2021), which could be cell-type specific. Supporting the aforementioned findings, experiments that inserted bacterial chromosomes with differing sequence composition into a *Saccharomyces cerevisiae* host identified changes in the bacterial chromosome 3D structure that were predictable from features derived from eukaryotic sequences, suggesting a strong influence of genome composition in driving 3D organisation (Chapard et al. 2022).

### Functional sequence motifs and elements

Through statistical modelling, as well as in silico and experimental perturbation studies, transcription factor motifs, cis-regulatory elements or CREs (enhancers, facilitators, insulators, promoters, ~ 8% of the human genome (Moore et al. 2020)), and novel functional sequences (e.g. early replicating control elements or ERCEs) have been linked to genome organisation from the level of nucleosomes to nuclear bodies.

Nucleosome-depleted regions are formed in regulatory sites, such as promoters and enhancers facilitating transcription (Chereji and Clark 2018). Intrinsic sequence composition can dictate nucleosome positioning, but the presence of sequence motifs recognised by nucleosome-competing proteins, can override intrinsic sequence signals like the tendency of GC-rich segments in promoters to form nucleosomes (Valouev et al. 2011; Chereji and Clark 2018). Pioneer transcription factors, for instance, can recognise motifs in compacted nucleosomal DNA, and their binding mediates remodelling and opening of the region (Slattery et al. 2014).

Sequence motifs and elements have also been associated with higher-scale architectural features. These include CTCF motifs, defined by degenerate 20-mer consensus motif in vertebrates (Kim et al. 2007; Plasschaert et al. 2014), housekeeping genes in TAD boundaries (Dixon et al. 2012), non-CTCF motifs for other insulators demarcating domain boundaries in flies (Ramírez et al. 2018), and contacts between enhancers and promoters. The absence of recently characterised facilitator elements, shown to augment enhancer activity, was shown to decrease the frequency of classical enhancer and promoter interactions (Blayney et al. 2023). Furthermore, linking replication timing to genome structure, early replicating control elements (ERCEs) were found to influence all levels of the intermediate-scale of organisation (Sima et al. 2019).

Significant portion of evidence for this feature category comes from recent deep-learning-based statistical models (based on human data if not specified otherwise) that allow base-pair level identification of regions most influential to the prediction of interactions and in silico perturbations. Findings from these models support the contribution of self and cross interactions of regulatory elements like promoters and enhancers (particularly those with proximal CTCF binding regions (Schwessinger et al. 2020)), the role of loop extrusion through the implication of RAD21 (RAD21 Cohesin Complex Component) and SMC3 (Structural Maintenance of Chromosomes 3) motifs, the significant organiser role of CTCF motifs and their orientation (Kelley et al. 2016; Trieu et al. 2020; Fudenberg et al. 2020; Schwessinger et al. 2020; Cao et al. 2021; Zhou 2022; Yang and Ma 2023; Tan et al. 2023; Al-jibury et al. 2023; Gunsalus et al. 2023), describing also the dependence of cell-type-invariant interactions on these motifs (Zhou 2022). Promoter-promoter, enhancer-promoter and enhancer-enhancer interactions were found to vary across cell types (Zhou 2022). Cell-type-specific interactions were linked to non-CTCF transcription factor motifs, such as the POU5F1(ATGCAAA)::SOX2(ACAATG) dimer motif and POU family motifs (ATGCAAAT) in H1 human embryonic stem cell (ESC) line, as well as the AP-1 or FOS::JUN (TGANTCA) dimer motif in HFF human foreskin fibroblast cell line, consistent with the cell-type-specific activity of transcription factors (Zhou 2022). With these models, authors did find in silico mutations outside these regulatory elements that change organisation (Fudenberg et al. 2020), predicted boundary sites independent of CTCF motifs (Trieu et al. 2020; Schwessinger et al. 2020), and driver elements not related to CTCF, known promoters or enhancers (Tan et al. 2023), collectively suggesting the existence of other sequence determinants. For instance, MYC-associated zinc finger or MAZ insulator sites (CCCCTCC, this and the motifs brought hereafter are based on consensus sequences from JASPAR 2024 (Rauluseviciute et al. 2024)), proximal or distal to CTCF sites, was found to contribute to the prediction of genome organisation (Tan et al. 2023) supporting earlier experiments associating MAZ with CTCF and cohesin organiser roles (Xiao et al. 2021). In *Drosophila*, another deep learning model predicting TAD boundaries found dependence on previously implicated insulator motif Beaf-32 (TATCGATA) (Ramírez et al. 2018), and on similar motifs Dref (TATCGA) and Pnr (ATCGAT) (Henderson et al. 2019). Supporting this, Hi-C analysis for “blocking” elements using generalised linear models highlighted Pnr along with Ttk (AGGANAA) as causative regulators (Mourad and Cuvier 2018). Later work has shown, however, that Beaf-32 and CTCF (GRTGGCGC in *Drosophila*) are not required for TAD formation in *Drosophila*, suggesting that this pattern might not be causative (Cavalheiro et al. 2023). At the compartment level, with a deep learning model designed to predict A/B compartmentalisation, A compartments were consistently negatively correlated with NANOG (GCAATCA in mouse), and PDX1 (ATTA in human, TAAT in mouse) and LHX3 (ATTAATT in mouse) homeoboxes in humans and mice, and strongly positively correlated with KLF4 (CACCC) transcription factor motif in humans (Prost et al. 2020). With the transformer-based model “UNADON”, promoters, enhancers and CTCF have also been shown to be predictive of the spatial location of regions relative to nuclear bodies across different cell types (Yang and Ma 2023), consistent with enrichment of CTCF binding sites in LAD borders (Harr et al. 2015).

### Repeat elements

The uneven distribution of repeat families has been correlated with genome organisation at different scales in multiple species. Transposable elements (TEs) have been known to spatially segregate into distinct regions (Korenberg and Rykowski 1988). B1/Alu- and L1/LINE1-rich sequences tend to self-segregate, forming clusters that are highly correlated with A/B compartments (Prost et al. 2020; Lu et al. 2021). Differences between organisms have been reported in this regard, such as LINE-1 showing a positive correlation with A compartment predictions in humans but a negative correlation in mice (Prost et al. 2020).

TEs are associated with domain boundaries across species, including humans (e.g. HERV-H) (Dixon et al. 2012; Zhang et al. 2019), rodents (B2 SINEs) (Kentepozidou et al. 2020; Choudhary et al. 2020), and fungi (Winter et al. 2018). They also provide insulator elements (e.g. MIRs) (Wang et al. 2015), which could block enhancer and promoter interactions and serve as barriers for domains. Repeats represent a significant amount of sequence, comprising around half (~ 56%) of the human genome (Smit et al. 2013), and transposons can be exapted into functional elements, such as CTCF sites (Schmidt et al. 2012), promoters and enhancers (Etchegaray et al. 2021), although relatively rare given their whole genome prevalence (Simonti et al. 2017).

In addition to functioning as insulators, the interactions of similar repeat elements (e.g. Alu elements and tandem repeats) have been linked to specific genomic contacts, such as enhancer-promoter interactions in humans, mice, and flies (Cournac et al. 2016; Gu et al. 2016; Nikumbh and Pfeifer 2017; Raviram et al. 2018; Bai et al. 2021), with complementary Alu sequences reported to determine enhancer-promoter pairing (Liang et al. 2023). Systematic in silico sequence perturbations using a deep learning model predicting contact maps from 1 Mb of DNA sequence demonstrated the importance of almost all repeat families. Notably, Alu, hAT-Charlie, small RNAs, and SVA showed impact, comparable to or greater than that of CTCF sites, and many of the most disruptive elements do not contain CTCF (Gunsalus et al. 2023). This work also showed that changes in GC content due to repeat insertion can influence genome folding. Finally, based on UNADON, satellite DNA, ERV-K long tandem repeats along with L1 elements and small RNA sequences are the top features for predicting cytological distance to nuclear bodies (Yang and Ma 2023).

### DNA secondary structures

DNA can, under certain conditions, deviate from its typical B form of the right-handed double helix structure (B-DNA), to form non-B secondary structures (tandem repeats prone to forming non-B DNA consist about ~ 3% of the human genome (Matos-Rodrigues et al. 2023)). Among these are R-loops (single-stranded RNA hybridises to one strand of a double-stranded DNA, displacing the other DNA strand), triplexes (typically formed by pairing of oligopurines and oligopyrimidines), G-rich G-quadruplexes (putative sequence (G_≥ 3_N_1−12_)_3_G_≥3_, ~ 716,000 sequences in humans (Chambers et al. 2015)), C-rich i-motifs (putative sequence (C_≥ 3_N_1–12_)_3_C_≥3,_ ~ 53,000 sequences in humans (Peña Martinez et al. 2024)), and Z-DNA (typically formed by alternating purine-pyrimidine repeats like GC/CG, 391 Z-DNA-forming sites in humans (Shin et al. 2016)), each associated with certain sequence patterns. These structures extend the functionality of nucleic acids beyond simple linear sequences, and their presence in cells has been detected using various methods (Biffi et al. 2013; Di Antonio et al. 2020; Matos-Rodrigues et al. 2023).

Non-B DNAs are key regulators of biological functions that can affect genome organisation across multiple scales, from nucleosome positioning, shorter-range loops within TADs to longer-range interactions mediating compartmentalisation, as evidenced by multiple studies. The enrichment of non-B DNAs, particularly Z-DNA (left‑handed parallel double helix) and G-quadruplexes (G4s, four-stranded DNA structure formed by stacked guanine tetrads), in nucleosome-depleted regions in *Caenorhabditis elegans*, yeast and human contexts (Wong et al. 2007; Halder et al. 2009; Wong and Huppert 2009; Hänsel-Hertsch et al. 2016) suggests that they block nucleosome formation. In addition, formation of non-B structures promotes or inhibits binding of transcription factors (Georgakopoulos-Soares et al. 2022), which could affect the interactions of genomic regions particularly involving regulatory elements.

Several studies implicate G4s and i-motifs in genome organisation. G4s alone can act as insulators, as well as reinforce insulation at G4-overlapping CTCF sites (Hou et al. 2019). Consistently, G4s bind to insulator protein CTCF in vitro (unlike i-motifs), potentially influencing CTCF recruitment (Tikhonova et al. 2021). They can on the other hand, directly bind with a ubiquitously expressed transcription factor, YY1, mediating enhancer-promoter interactions and facilitating gene regulation *via* DNA looping (Li et al. 2021), consistent with the observed enrichment of G4-prone sequences and G4 ChIP-seq peaks at promoters and distal CREs like enhancers (Hegyi 2015; Hou et al. 2019). RNA polymerase II-mediated loops were also found to be correlated with G4 locations based on G4 ChIP-seq (Yuan et al. 2023). I-motifs (formed by intercalated cytosine-cytosine base pairs) also significantly overlap with CREs and open chromatin regions, suggesting regulatory roles (Ma et al. 2022; Zanin et al. 2023). Subsets of TAD boundaries also contain i-motifs (Peña Martinez et al. 2024) and G4s (Hou et al. 2019). G4-containing boundaries have higher degree of architectural protein binding (than non-G4 ones), potentially explaining their observed stronger interactions (Hou et al. 2019).

Beyond DNA-only structures, RNA-mediated non-B configurations have been found to influence TAD architecture and genome compartmentalisation. R-loops mediated by lncRNA (i.e. HOTTIP) can reinforce CTCF-bound TAD boundaries, supported by experiments eliminating these loops (Luo et al. 2022). On one hand, lncRNA-DNA triplexes are enriched in TADs and have been used to discriminate TADs from other genomic regions (Soibam and Zhamangaraeva 2021). LncRNA GATA6-AS1 triplex sites were found to be enriched in TAD boundaries, computationally predicted to bind CTCF and located in areas that undergo TAD reorganisation with cardiac differentiation of human embryonic stem cells (Soibam 2022). With MD simulations, it was shown that co-localisation of RNA-mediated triplexes could potentially contribute to genome organisation, primarily influencing longer-range interactions underlying A/B compartmentalisation (Farabella et al. 2021).

## Discussion

In this review, we have summarised the sequence-based features linked to 3D genome organisation, providing an emerging perspective of the sequence code of genome architecture. This work underscores that different types of sequence features can play a role at multiple scales of genome organisation, with that role strongly influenced by the main mechanism of organisation at a given scale, and the cell context due to the cell-state specificity of some sequence features, such as the usage of regulatory elements and transcription factor binding sites. Moving forward, it would be valuable to systematically evaluate and compare the importance of these interpretable sequence features across different scales and contexts, while also considering their interrelatedness. For example, GC-rich sequences tend to be enriched in motifs forming G-quadruplex structures, the nucleotide composition of different repeat elements are specifically biased, and regulatory elements, like enhancers and promoters, could have in part originated from exapted repeat elements. In addition, contributions of fundamental sequence-derived features inherent to the sequence, such as k-mer associated thermodynamic and quantum mechanical properties (Masuda et al. 2023), should be explored. It would be interesting to model 3D genome organisation based on these intrinsic sequence features and then compare the results with experimental data from different cell types or states. These comparisons would help determine how well these sequence-based models recapitulate genome organisation and pinpoint the scales at which sequence features exert the greatest influence.

Finally, we encourage experimental investigations to validate and disentangle the potential direct and/or indirect contributions of genomic sequence to the genome organisation, and its interplay with earlier-characterised protein and epigenetic determinants. For example, while sequence elements can serve as indirect organisers by binding protein drivers, in silico (Mazur 2016), in vitro (Wang et al. 2010) and in vivo (Gladyshev and Kleckner 2014) mechanistic studies of DNA duplex association (Mazur et al. 2020), and phase separation mechanisms (Erdel and Rippe 2018) support potential direct mechanisms of contribution. In vitro studies have already demonstrated the preferential association of identical DNA duplexes. Similar experimental setups could be leveraged to determine whether certain sequence features would indeed influence these associations using strategic oligonucleotide designs. Bound proteins, complementary RNAs, sequence modifications already shown to be important drivers of associations between genomic regions could then be added to the system to test how they alter the patterns and degree of associations, with the goal of disentangling the contribution of sequence from non-sequence factors. Readouts measuring the effect on genomic interactions can then be obtained using a variety of methods, such as higher-resolution chromosome conformation capture, imaging, biophysical techniques, or in vivo looping assays (Hao et al. 2021).

## Data Availability

No datasets were generated or analysed during the current study.
